# Supplementary material for: Mechanism of interaction of an endofungal bacterium Serratia marcescens D1 with its host and non-host fungi
Source: PLoS One. 2020 Apr 22;15(4):e0224051. doi: 10.1371/journal.pone.0224051 (PMC7176118; doi:10.1371/journal.pone.0224051)
Supplement: S1 Fig — A. Amplification of 16S rRNA gene in the screened cultures. Lane 1: 1 kb DNA ladder; Lane 2–8: sample IDs SS7, OR4.1, AAU-R4, AAU-R6, SC2.2, SC4.6 and HB8, respectively; Lane 9: sample SS1 showing no amplification for 16S rRNA gene; Lane 10: Escherichia coli K12 as positive control for amplification. B. Amplification of 16S rRNA gene from the total genomic DNA isolated after two subsequent subcultures of the positive isolates. Lane 1: 1 kb DNA ladder; Lane 2–8: sample IDs SS7, OR4.1, AAU-R4, AAU-R6, SC2.2, SC4.6 and HB8, respectively; Lane 9: Escherichia coli K12 as positive control for amplification. (DOCX) [file pone.0224051.s001.docx]

**
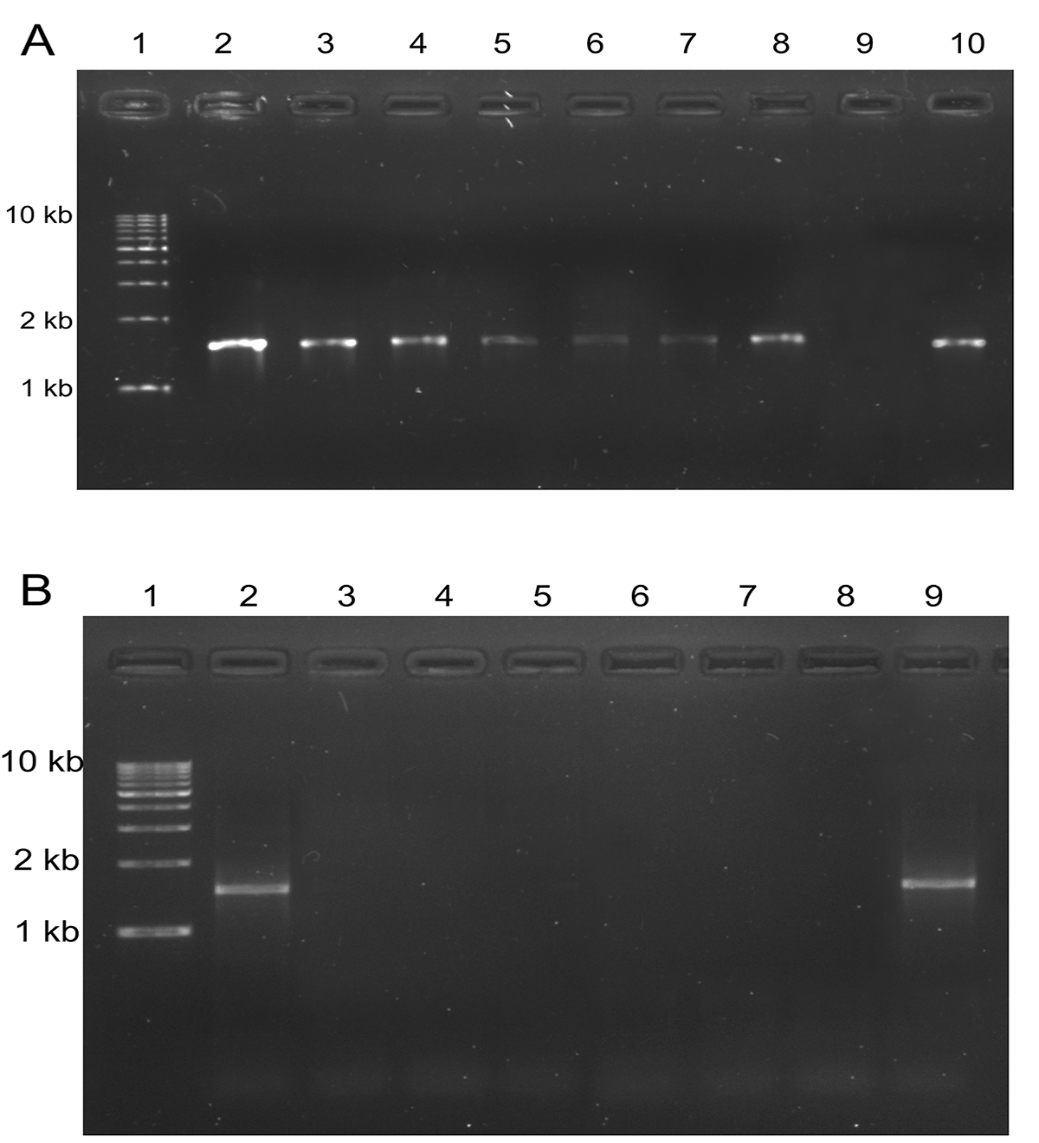
**

**Figure S1: PCR based screening of the presence of bacterial 16S rRNA gene in the total genomic DNA isolated from the fungal cultures. A.** Amplification of 16S rRNA gene in the screened cultures. Lane 1: 1 kb DNA ladder; Lane 2-8: sample IDs SS7, OR4.1, AAU-R4, AAU-R6, SC2.2, SC4.6 and HB8, respectively; Lane 9: sample SS1 showing no amplification for 16S rRNA gene; Lane 10: *Escherichia coli* K12 as positive control for amplification. B. Amplification of 16S rRNA gene from the total genomic DNA isolated after two subsequent subcultures of the positive isolates. Lane 1: 1 kb DNA ladder; Lane 2-8: sample IDs SS7, OR4.1, AAU-R4, AAU-R6, SC2.2, SC4.6 and HB8, respectively; Lane 9: *Escherichia coli* K12 as positive control for amplification.
