# Supplementary figures and images for: Mechanism of interaction of an endofungal bacterium Serratia marcescens D1 with its host and non-host fungi
Source: PLoS One. 2020 Apr 22;15(4):e0224051. doi: 10.1371/journal.pone.0224051 (PMC7176118; doi:10.1371/journal.pone.0224051)

**­­­­
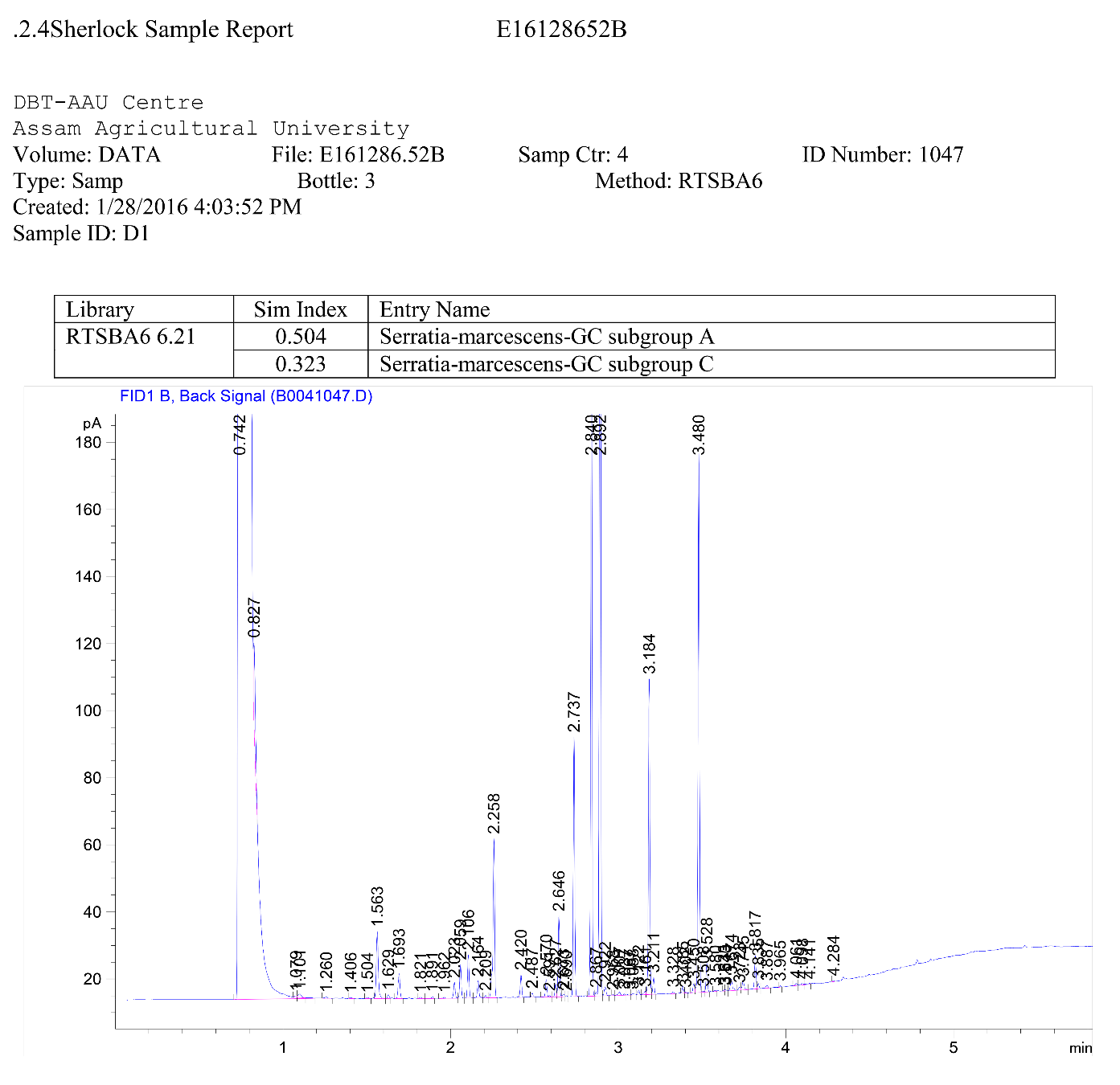
**

**Figure S2: FAME analysis report showing similarity index of isolate D1.**

Supplement: S2 Fig — (DOCX) [file pone.0224051.s002.docx]
