# Supplementary material for: Mechanism of interaction of an endofungal bacterium Serratia marcescens D1 with its host and non-host fungi
Source: PLoS One. 2020 Apr 22;15(4):e0224051. doi: 10.1371/journal.pone.0224051 (PMC7176118; doi:10.1371/journal.pone.0224051)
Supplement: S3 Fig — A. hyphae of the fungal isolate showing rhizoids; B. A young sporangia on the terminal hyphae; C. spores of the fungal isolates; scale bar = 10 μm. (DOCX) [file pone.0224051.s003.docx]

**
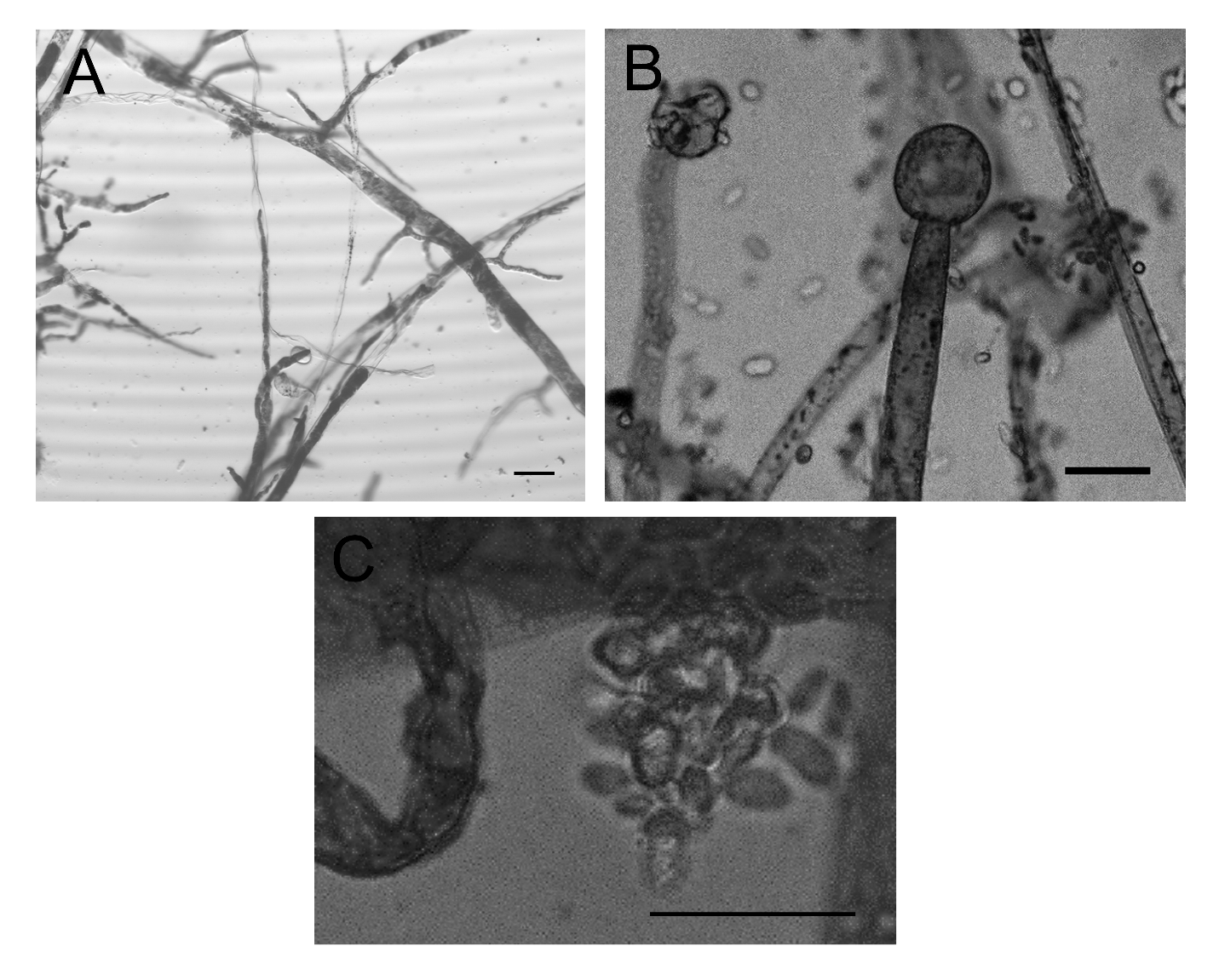
**

**Figure S3: Microscopic observations of the fungal hyphae and spores of the fungal isolate SS7. A.** hyphae of the fungal isolate showing rhizoids; **B.** A young sporangia on the terminal hyphae; **C.** spores of the fungal isolates; scale bar = 10 μm.
