# Supplementary material for: Mechanism of interaction of an endofungal bacterium Serratia marcescens D1 with its host and non-host fungi
Source: PLoS One. 2020 Apr 22;15(4):e0224051. doi: 10.1371/journal.pone.0224051 (PMC7176118; doi:10.1371/journal.pone.0224051)
Supplement: S4 Fig — Mucor irregularis SS7 was used as positive control. Photographs were taken after 48 h of bacterial interaction with the fungal hyphae. Pink red pigmentation indicated bacterial spreading. (DOCX) [file pone.0224051.s004.docx]

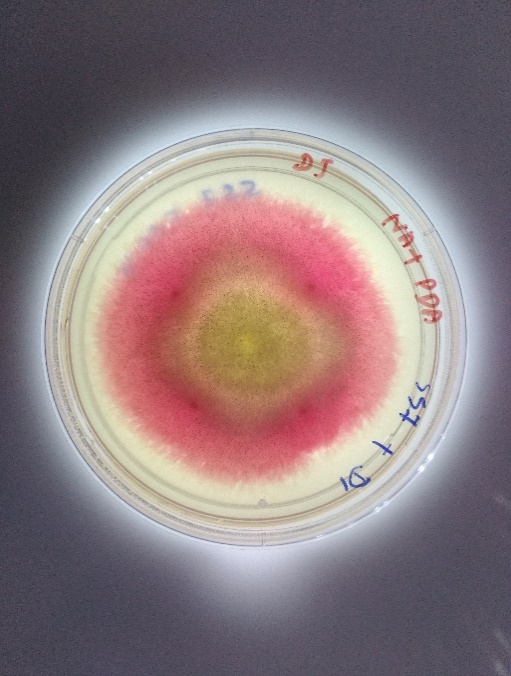

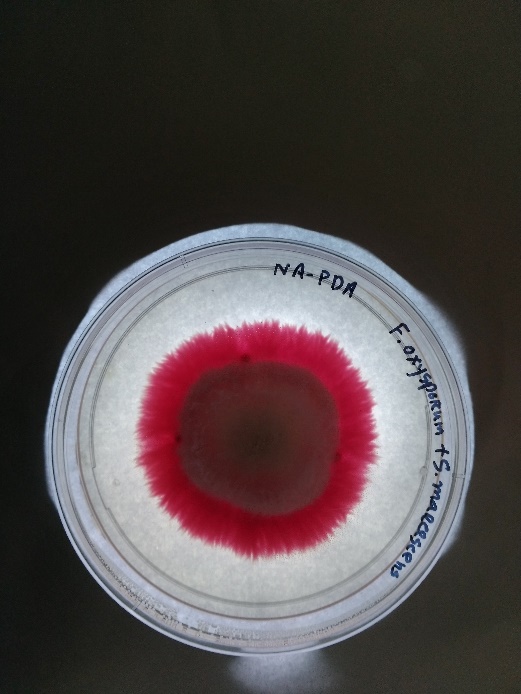

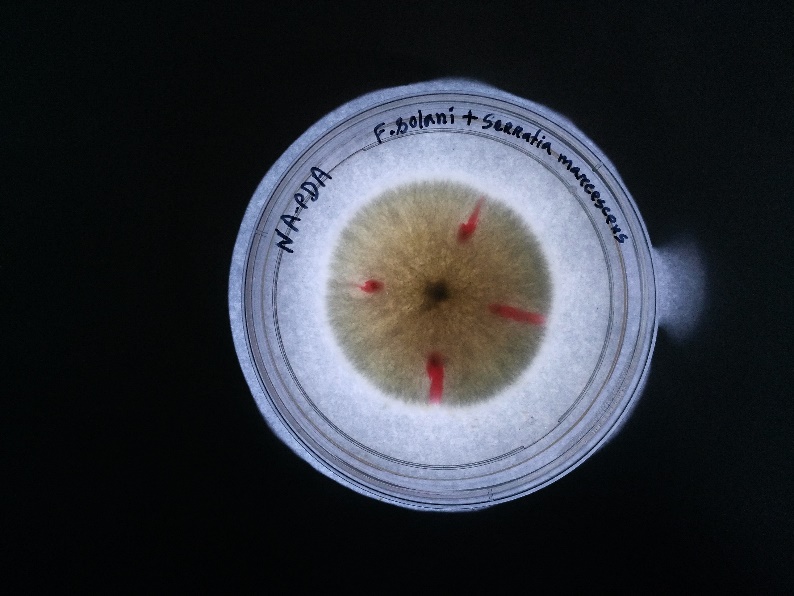

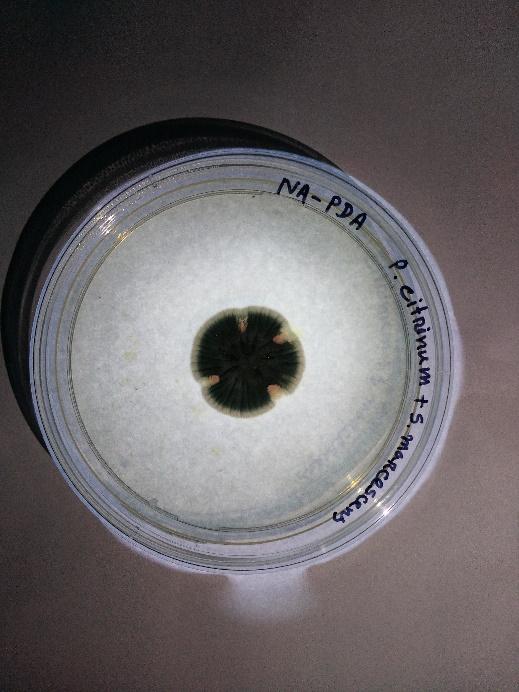

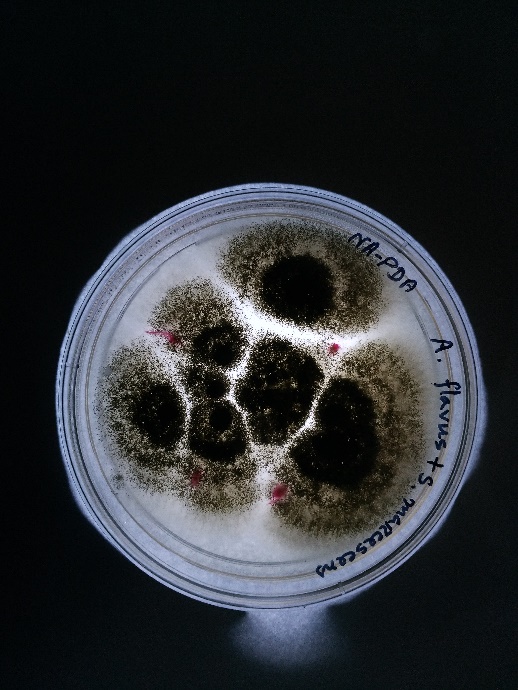

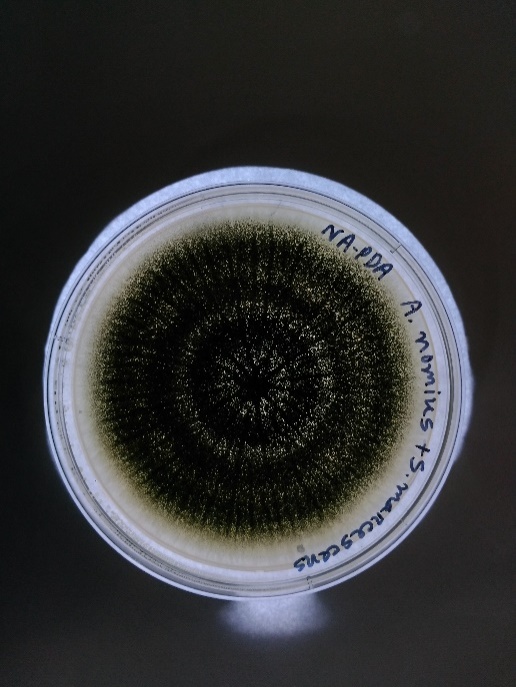


*Aspergillus nomius* F12

*Aspergillus flavus* F16

*Fusarium solani* F8

*Fusarium oxysporum* SC7.1

*Penicillium citrinum* F14

*Mucor irregularis* SS7

**Figure S4: Interaction of *Serratia marcescens* with Ascomycetes fungi.** *Mucor irregularis* SS7 was used as positive control. Photographs were taken after 48 h of bacterial interaction with the fungal hyphae. Pink red pigmentation indicated bacterial spreading.
