# Supplementary material for: Mechanism of interaction of an endofungal bacterium Serratia marcescens D1 with its host and non-host fungi
Source: PLoS One. 2020 Apr 22;15(4):e0224051. doi: 10.1371/journal.pone.0224051 (PMC7176118; doi:10.1371/journal.pone.0224051)
Supplement: S5 Fig — Photographs were taken after 48 h of bacterial interaction with the fungal hyphae. Pink red pigmentation indicated bacterial spreading. (DOCX) [file pone.0224051.s005.docx]

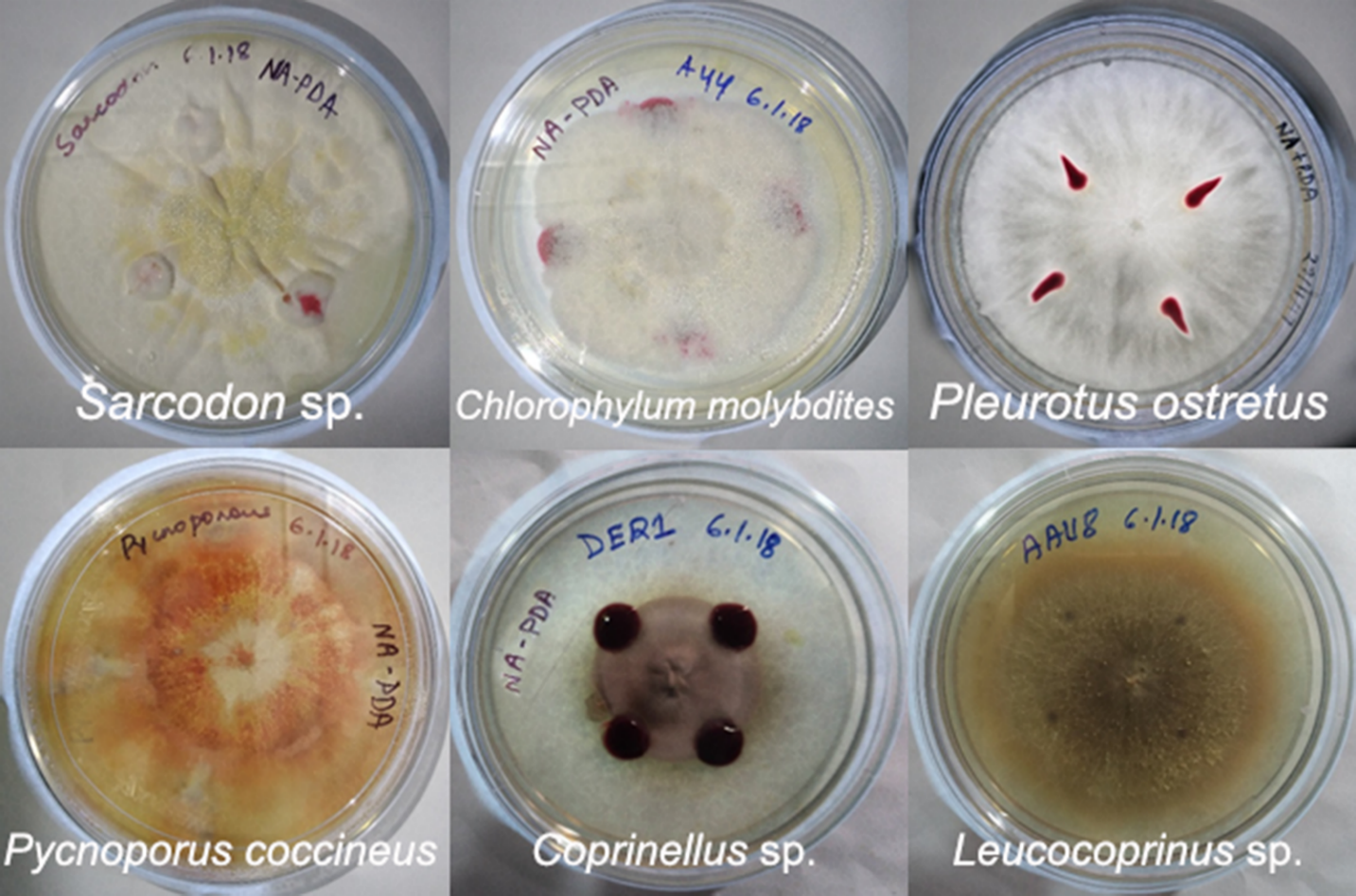


**Figure S5: Interaction of *Serratia marcescens* with Basidiomycetes fungi.** Photographs were taken after 48 h of bacterial interaction with the fungal hyphae. Pink red pigmentation indicated bacterial spreading.
