# Supplementary material for: Mechanism of interaction of an endofungal bacterium Serratia marcescens D1 with its host and non-host fungi
Source: PLoS One. 2020 Apr 22;15(4):e0224051. doi: 10.1371/journal.pone.0224051 (PMC7176118; doi:10.1371/journal.pone.0224051)
Supplement: S6 Fig — Error bars represents the standard deviations of three independent replications. (DOCX) [file pone.0224051.s006.docx]

**
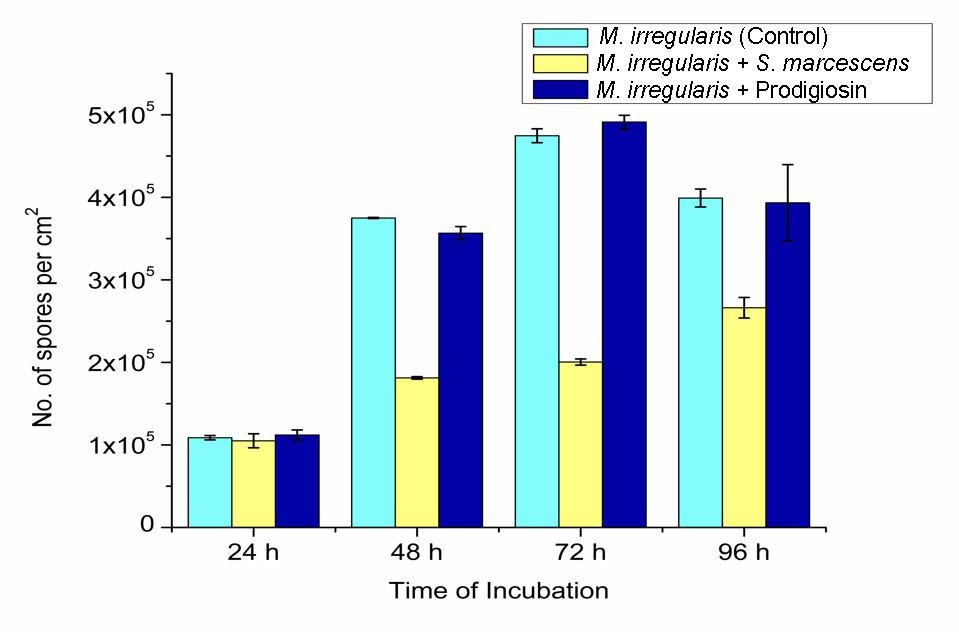
**

**Figure S6: *M. irregularis* SS7 spore count at different time interval after treatment with *S. marcescens* D1 and its pink-red pigment prodigiosin.** Error bars represents the standard deviations of three independent replications.
