# Supplementary material for: Mechanism of interaction of an endofungal bacterium Serratia marcescens D1 with its host and non-host fungi
Source: PLoS One. 2020 Apr 22;15(4):e0224051. doi: 10.1371/journal.pone.0224051 (PMC7176118; doi:10.1371/journal.pone.0224051)
Supplement: S7 Fig — Upper rows contains the brighfield images of the fungal hyphae. Lower row shows the images with red fluorescence of propidium iodide. (DOCX) [file pone.0224051.s007.docx]

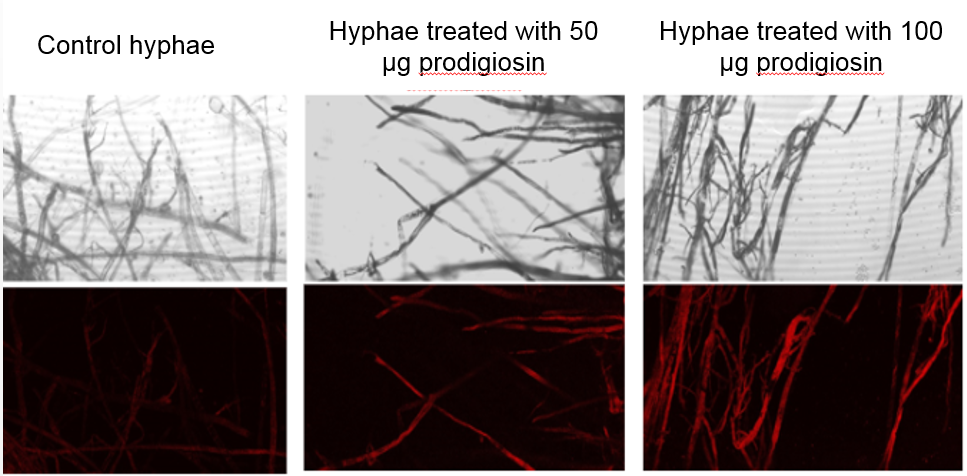


**Figure S7: Pore formation on cell membrane of *Mucor irregularis* SS7 detected by the intensity of propidium iodide with increasing concentrations of prodigiosin.** Upper rows contains the brighfield images of the fungal hyphae. Lower row shows the images with red fluorescence of propidium iodide.
