# Supplementary material for: Mechanism of interaction of an endofungal bacterium Serratia marcescens D1 with its host and non-host fungi
Source: PLoS One. 2020 Apr 22;15(4):e0224051. doi: 10.1371/journal.pone.0224051 (PMC7176118; doi:10.1371/journal.pone.0224051)
Supplement: S1 Table — (DOCX) [file pone.0224051.s008.docx]

**Table S1: Primers designed for quantitative real-time PCR analysis**

| **Gene** | **Primer** | **Sequence (5‘ - 3‘)** | **Length** |
| --- | --- | --- | --- |
| *pigC* | qPigC-F | CCTGGTGGAGACGGTCAATC | 20 |
|  | qPigC-R | TCAATTCGAACTCCTGGCGG | 20 |
| *pigF* | qPigF-F | GGATGGATGCTGCACGACTA | 20 |
|  | qPigF-R | GGTGGAGACCAGCATGTTCA | 20 |
| *pigM* | qPigM-F | CTCTCCACTATGACGCCGAC | 20 |
|  | qPigM-R | GCGCTGAGGTACTCGAAGAA | 20 |
| *copA* | qCopA-F | CCGGTTATCAGGCCGAAGTC | 20 |
|  | qCopA-R | CATACACTTTCGCGCTGTCG | 20 |
| *cueR* | qCueR-F | ACGGTTACCGCCACTATTCC | 20 |
|  | qCueR-R | CTTTTCGATCTCCGCCACCT | 20 |
| *murLP* | MurLP-F | ATCCTGGGTTCCACTCTGCT | 20 |
|  | MurLP-R | GCAGCTTGAACGTCAGAACG | 20 |
| *tssJ* | TssJ-F | TACAGCCTGGTGTTCGATGC | 20 |
|  | TssJ-R | TTTGGCGTCGTTTTGCAGAC | 20 |
| *chiA* | ChiA-F | TCGTCGGTTCGGTGAAAGAG | 20 |
|  | ChiA-R | AGCAGCACATAGGTTTCCCC | 20 |
| *gyrB* | GyrB-F | AAACTGGAGCTGGTGATCCG | 20 |
