# Supplementary material for: Mechanism of interaction of an endofungal bacterium Serratia marcescens D1 with its host and non-host fungi
Source: PLoS One. 2020 Apr 22;15(4):e0224051. doi: 10.1371/journal.pone.0224051 (PMC7176118; doi:10.1371/journal.pone.0224051)
Supplement: S2 Table — (DOCX) [file pone.0224051.s009.docx]

**Table S2: Morphological and biochemical test results for the bacterial isolate D1**

| **Test name** | **Result** |
| --- | --- |
| Gram’s staining | Gram negative |
| Cell shape | Rod |
| Colony morphology (on nutrient agar) | Round; red (25°C), light-pink (30°C), white (37°C) |
| Spore | Negative |
| Pigment | Positive |
| Catalase | Positive |
| Gelatin hydrolysis | Positive |
| Motility | Positive |
| Indole | Negative |
| Methyl red | Negative |
| Voges-Proskauer | Positive |
| Citrate | Positive |
| Nitrate reduction | Negative |
| Urease | Negative |
| ONPG | Positive |
| Acid production from glucose | Positive |
| Oxidase | Negative |
| **Sugar utilization** | |
| Cellobiose | Negative |
| Fructose | Positive |
| Galactose | Positive |
| Glucose | Positive |
| Glycerol | Positive |
| Inositol | Positive |
| Lactose | Negative |
| Maltose | Positive |
| Mannitol | Positive |
| Mannose | Positive |
| Raffinose | Negative |
| Rhamnose | Negative |
| Ribose | Positive |
| Sorbitol | Positive |
| Sucrose | Positive |
