# Supplementary material for: Mechanism of interaction of an endofungal bacterium Serratia marcescens D1 with its host and non-host fungi
Source: PLoS One. 2020 Apr 22;15(4):e0224051. doi: 10.1371/journal.pone.0224051 (PMC7176118; doi:10.1371/journal.pone.0224051)
Supplement: S3 Table — (DOCX) [file pone.0224051.s010.docx]

**Table S3: Results of the antibiotic susceptibility test for the bacterial isolate D1**

| **Antibiotic** | **Code** | **Concentration**  **(µg)** | **Diameter of zone of inhibition ± SD^#^**  **(mm)** | **Susceptibility** |
| --- | --- | --- | --- | --- |
| Amoxyclav | AMC | 30 | - | Resistant |
| Ampicillin | AMP | 10 | - | Resistant |
| Cephalothin | CEP | 30 | - | Resistant |
| Cefoxitin | CX | 30 | 21.67 ±4.04 | Susceptible |
| Ceftazidime | CAZ | 30 | 18.33 ±1.53 | Susceptible |
| Chloramphenicol | C | 25 | 17.67 ±2.08 | Susceptible |
| Clindamycin | CD | 2 | 16.33 ±0.58 | Susceptible |
| Erythromycin | E | 15 | 13.00 ±1.00 | Susceptible |
| Gentamycin | GEN | 10 | 23.00 ±0.00 | Susceptible |
| Ofloxacin | OF | 5 | 30.00 ±1.00 | Susceptible |
| Oxacillin | OX | 1 | - | Resistant |
| Penicillin | P | 1 units, 10 units | - | Resistant |
| Streptomycin | S | 10 | 21.33 ±2.52 | Susceptible |
| Sulphatriad | S3 | 300 | 21.33^*^ ±1.53 | Resistant^*^ |
| Tetracyclin | TE | 25 | 19.00 ±3.00 | Susceptible |
| Teicoplanin | TEI | 30 | 18.67 ±1.15 | Susceptible |
| Vancomycin | VA | 30 | 13.00 ±1.73 | Susceptible |
| ^#^All the data were represented as the average ± standard deviation (SD) of minimum three biological replicates. *Bacterial cells were resistant, but red pigmentation was completely inhibited within the zone of action of the antibiotic. | | | | |
