# Supplementary material for: Mechanism of interaction of an endofungal bacterium Serratia marcescens D1 with its host and non-host fungi
Source: PLoS One. 2020 Apr 22;15(4):e0224051. doi: 10.1371/journal.pone.0224051 (PMC7176118; doi:10.1371/journal.pone.0224051)
Supplement: S4 Table — (DOCX) [file pone.0224051.s011.docx]

**Table S4: Inhibitory effect of prodigiosin on fungal cultures.**

| **Sl No** | **Test Fungi** | **Inhibition percentage (±SD)** |
| --- | --- | --- |
| 1 | *Mucor irregularis* SS7 | 28.59±2.2 |
| 2 | *Fusarium oxysporum* SC7.1 | 37.19±1.8 |
| 3 | *Fusarium solani* F8 | 32.40±3.5 |
| 4 | *Aspergillus flavus* F16 | 17.18±3.3 |
| 5 | *Aspergillus nomius* F12 | 18.87±1.6 |
| 6 | *Pennicillium citrinum* F14 | 16.46±1.9 |
